# Serial casting for contractures in SMA: consensus derived guidelines for treatment

**DOI:** 10.3389/fneur.2025.1502495

**Published:** 2025-04-16

**Authors:** Laurey Brown, Katie Hoffman, Chiara Corbo-Galli, Carolyn Kelley, Terri Carry, Matt Civitello, Giorgia Coratti, Roberto DeSanctis, Tina Duong, Brigid Driscoll, Jean Flickinger, Allan M. Glanzman, Jennifer Jones, Elizabeth Maczek, Dionne Moat, Jacqueline Montes, Robert Muni-Lofra, Leslie Nelson, Amy Pasternak, Melanie Valle, Kristin J. Krosschell

**Affiliations:** ^1^Ann & Robert H. Lurie Children's Hospital of Chicago, Chicago, IL, United States; ^2^Weinberg College of Arts and Sciences, Northwestern University, Evanston, IL, United States; ^3^Children's Hospital of Colorado, Aurora, CO, United States; ^4^St. Jude Children's Research Hospital, Memphis, TN, United States; ^5^Agostino Gemelli University Hospital, Rome, Italy; ^6^Department of Neurology, Stanford University, Stanford, CA, United States; ^7^Children's Hospital of Philadelphia, Philadelphia, PA, United States; ^8^Boston Children's Hospital, Boston, MA, United States; ^9^Newcastle Upon Tyne Hospitals NHS Foundation Trust, Newcastle upon Tyne, United Kingdom; ^10^Columbia University Irving Medical Center, New York, NY, United States; ^11^Dallas Children’s Medical Center, Dallas, TX, United States; ^12^Department of Physical Therapy, University of Texas – Southwestern, Dallas, TX, United States; ^13^Northwestern University Feinberg School of Medicine, Chicago, IL, United States

**Keywords:** Delphi technique, consensus, spinal muscular atrophy, contracture, serial casting

## Abstract

**Background:**

Individuals with Spinal Muscular Atrophy (SMA) often present with muscle contractures. Serial casting has been used in a variety of other peripheral nerve, muscle, and central nervous system disorders to improve knee and ankle range of motion limitations and functional performance in both ambulatory and non-ambulatory individuals.

**Objective:**

The goal of this study was to reach a consensus about the parameters, considerations and general guidelines that should inform practice when serial casting to improve flexibility in individuals with SMA.

**Methods:**

This international effort was conducted between August 2020 and May 2023. An expert panel of physical therapists was assembled with multiple panel meetings and a 2-round Delphi survey performed covering topics relevant to serial casting which included three domains: clinical appropriateness, program-based considerations and program adherence/feasibility. Consensus was reached for all items in the three topic areas using a validity index of >75%. Following completion of the Delphi survey, community insights from patients and caregivers were collected via semi-structured interview.

**Results:**

This study included the synthesis of meetings from the initial expert panel which produced a comprehensive survey for the different considerations when performing serial casting for an individual with SMA. The study also included the completion of a Delphi survey by 18 therapists in round 1 and 15 therapists in round 2 for a consensus on 296 items. Strong consensus was obtained in all three domains with 96.6% agreement for clinical appropriateness, 95.0% agreement for program-based considerations, and 96.9% for program adherence/feasibility. A guideline document was developed enumerating the specific items detailed in the survey. Community perspectives were utilized to support the results of the Delphi survey and to add insight into real-world experience.

**Conclusion:**

The serial casting guidelines developed upon collaborative discussions from the expert panel and from the Delphi survey consensus and semi-structured interviews, should be utilized when applying serial casts to patients with spinal muscular atrophy. Future endeavors should look to apply the guideline recommendations to determine casting efficacy for improving joint contracture and impact on function for those with SMA.

## 1 Introduction

Spinal muscular atrophy (SMA) is a progressive neuromuscular disease that results in global weakness and limitations in functional performance. SMA impacts the anterior horn cell, skeletal muscles, liver, pancreas, spleen, blood vessels, and the heart (1). Progressive joint contractures is one of the hallmark impairments in SMA which severely limit a person’s ability to perform certain tasks despite gains in strength (2). This impairment persists despite the advent of disease modifying therapies.

Contracture presence varies based on the individual’s type of SMA. Hip and knee extension limitations greater than 20 degrees are observed in 22–50% of those with Type II (3), and ankle dorsiflexion is limited in80% of those with Type III (2). Others (Salazar and Fujak refs here) demonstrate significant range impairments starting in early childhood and increasing with age.

SMN protein is found in skeletal muscle, contributes to disease pathology and is transcribed primarily from the SMN1 gene and to a limited extent from the SMN2 gene. In typical conditions, the SMN1 gene is the primary source of full length SMN protein, but in individuals with SMA, no SMN1 is present, however a significantly reduced functional SMN (~10%) protein is made by each copy of SMN2 (1). More copies of SMN2 are typically associated with diminished severity of SMA and have been associated with better functional outcome (1). SMN deficiency results in defects in individual myofibers, neuromuscular junctions, muscle dysfunction and overall survival (4). While SMN protein has an essential impact on muscle performance, there is, “no obvious function, pathway or set of factors that connect SMN uniquely to the health of the muscle (5).” SMN2 copies have a role in ensuring muscle growth through certain periods of life but are unable to sustain myofiber health for the lifespan. There is also evidence that different muscles are more vulnerable to low SMN with observed myopathy increased in muscles that have more sustained activity (5).

While the presence of joint contracture is widely accepted as a common impairment, there is a paucity of understanding of muscle architecture in SMA and how changes in muscle might relate to contracture formation. There is suspicion that a primary defect actually occurs at the level of the muscle (5). Studies highlight elevated levels of creatine kinase and a disorganization of myofibrils, sarcomeres and filaments across SMA types (5). Explorative studies have investigated low SMN levels and observed the disruption of myogenesis as well as the presence of myoblast fusion defects. Additional studies using the fly model have observed the profound alteration of thoracic muscle morphology and gone on to demonstrate the interaction and colocalization of SMN with myofibrillar *α*-actinin (6). SMN is known to be a sarcomeric protein that creates a complex with α-actinin and is localized in Z-discs in Drosphilia and mouse myofibrils (7). In humans, SMN is localized to the I-band and the M-band of the sarcomere, which suggests it may play a role in the sacromere’s structure. In SMA type I, the loss of SMN results in both atrophic and hypertrophic myofibers with alterations of Z-disc formation and actin filaments present. Unlike previous studies on Drosphilia models, human SMA localizes in the M-band and forms granules “flanking” the Z-disc, as opposed to being in the actual Z-disc, itself (4).

Regardless of emerging literature and suggestive contributions of SMN and the role it plays in muscle and muscle development, the mechanisms and underlying muscle pathology in SMA are poorly understood which challenges providers seeking best treatments for resulting phenotypic presentations and the associated impairments affecting muscle, specifically contractures and altered muscle length.

However, acknowledging a pathological presence at the level of the sarcomere can initiate a consideration for interventions at this level. In a study investigating sarcomere adaptations, Williams and Goldspink (8) found that during postnatal growth, increased muscle length resulted from the serial addition of sarcomeres with stimulus including increased bone length and movement of muscle through a typical range. This study noted that when newborn muscles are immobilized in extended positions, sarcomeres are reduced.

In order to provide immobilization in extension that could allow for improved muscle length with true sarcomere addition, serial casting has been applied as a potential strategy to modify/reduce the presence of contractures in a variety of disorders, including SMA (9). Serial casting uses a series of lower leg plaster casts applied successively and progressively over a period of time in order to immobilize a joint and hold the musculotendon unit in relative stretch with the goal of improving a joint’s mobility Casting supplies a form of static, low load stretch over prolonged periods of time (10). When a muscle is held in a relatively shortened position a subsequent loss of sarcomeres leads to shortening and the muscle fibers then adapt with a decreased length (11). One might hypothesize that through casting, growth of fiber length is promoted through serial sarcomerogenesis by building new contractile material in series with the muscle tendon unit. This then allows the muscle the opportunity to improve range of motion and extensibility (12). The overall goal of casting is to increase passive range of motion in the interest of promoting an individual’s highest level of function and mobility (13).

In animal models, muscle lengthening was demonstrated with an increase in the number of sarcomeres and an increase in tendon extensibility following immobilizations in elongated positions using casts (14). There are no noted changes in fascicle and muscle extensibility nor muscle belly or tendon length, but the improved tendon extensibility is noted to increase by about 20% (10). Conversely, immobilization in positions of shortened muscle length leads to a loss of sarcomeres, an increase in muscle tension, and profoundly decreased stretch (14, 15).

There is no direct evidence to support serial casting in SMA, however casting has been used to treat joint contractures present in other neuromuscular diagnoses such as DMD and Charcot Marie Tooth Disease (CMT) (16–18) and a general evidence-based care guideline for management of serial casting for those with cerebral palsy (CP), traumatic brain injury (TBI), DMD, and idiopathic toe walking (ITW) and spasticity has been published (19). This guideline highlights that in the presence of decreased range of motion at a joint, there is abnormal movement and alignment and subsequently decreased functional ability. When used in DMD, casting yields an increase in calf muscle length and no loss of function or strength (16). Casting outcomes in DMD have also demonstrated an associated stability on timed tests as well as stable gait function and speed with the improvements in ROM (17). Casting in CMT has demonstrated improved ankle range of motion and performance on 10 m walk test when applied for 4 weeks (18).

When applied to individuals with CP, serial casting is noted to improve the range of motion at the ankle and has a positive effect on function as measured by the Gross Motor Function Measure. However, it does not appear to change an individual’s gait velocity or stride length. CP typically presents with concerns of spasticity, which is not a characteristic of SMA, thus limiting the generalizations that can be made when comparing serial casting in both groups. The specific concerns regarding spasticity should be acknowledged, however, as spastic muscle fibers have notably long sarcomeres which may prevent additional sarcomeres from forming (20). In one study, negative effects of casting were reported, including difficulty bathing at home and dissatisfaction with the procedure. Other potential adverse effects of casting included foot pain, calf pain, skin inflammation, pressure sores and lower limb atrophy (19). One must also consider that variations in casting protocols and post-casting follow through may affect outcomes.

Casting has been presented as a less costly, less stressful, and less invasive alternative to surgical interventions such as achilles tendon lengthening, with casting demonstrating as much efficacy in range improvement as surgical lengthening (17). For CMT when non-invasive casting strategies are applied, surgical intervention is avoided or delayed by up to 4.5 years allowing for appropriate time for growth and continued mobility (21). Additional evidence demonstrates a loss of strength of one manual muscle test grade in individuals who have undergone surgical lengthening, which provides additional support for conservative management (22) Furthermore, serial casting for one limb over 4 weeks costs less than one-half of the surgical costs and avoid spotential risks such as infection, scarring and effects from anesthesia (19).

Though serial casting is a potentially effective conservative strategy for contracture management, challenges to note and consider at the outset of creating a serial casting process guideline include: variability in patient response; concerns for skin integrity; patient cognitive ability and ability to communicate; variation in materials; variation in length of casting procedures and variation in casting procedures, themselves (19). While serial casting is cost-effective and safe for managing contractures, given the lack of direct evidence and standardization of procedures for serial casting in SMA, a standardized process needs to be established before efficacy can be fully examined under controlled clinical conditions. Consensus based guidelines could improve care, functional outcomes, and patient/family satisfaction and decrease unwarranted variation in care and the need for invasive procedures (19). Using categories of clinical appropriateness, program-based considerations and program adherence/feasibility, this study sought to identify specific criteria for each category based on the consensus opinion of an international panel of expert clinicians. Consensus was then expanded to include a larger group of therapists representing practices in the United States and in Europe. Finally, community perspectives were added to ensure the guidelines reflected a real-world experience and would be realistic to the SMA community.

## 2 Methods

An expert panel composed of 18 physical therapists were selected based on their experience and knowledge in the field of SMA. While other provider types were initially considered, given the background, scope of practice and expertise of physical therapists for movement impairments, this provider group was thought most capable of providing recommendations. There were two representatives chosen for each expert site in the US with representation from the East and West Coasts and Midwest and Southern regions. The lead site had one additional therapist representative and one panel member with licensure in both physical therapy and orthotics. European panel members were selected based on interest and availability and ultimately included one representative from the UK and one from Italy.

Three initial meetings of the expert panel were held virtually prior to the creation and matriculation of the Delphi survey. The first two meetings reviewed the proposed domains and associated list of questions by the lead site with discussions concerning each area. The discussions were recorded and summarized following both meetings with additions, modifications, and recommendations made to the developing list of questions. The third meeting consisted of case study reviews from three different panel member locations. The outcome of these meetings was the creation of the first round of the Delphi survey.

A two-round on-line modified Delphi survey was utilized to obtain a consensus on criteria necessary for successfully performing serial casting on individuals with SMA. The Delphi method was initially developed by Dalkey (23) and is commonly utilized in health science research as a reliable way to reach a consensus on areas of clinical concern. The Delphi method involves both a working group and a participant group.

Between rounds one and two of the survey, the expert panel (working group) convened again with two optional virtual meetings to review the results of the initial round. Discussions were held in the areas where there was not agreement with recommendations made for survey wording. From these discussions, the second-round survey was developed.

RedCap, a secure web application for building and managing online surveys and databases, was used for conducting the survey rounds online (Vanderbilt University, Nashville, TN, USA).

Patient and parent interviews were conducted via semi-structured strategy. Interviews were conducted over Zoom with conversations recorded for analysis and extraction of important information.

This study was approved by the International Review Board (IRB) (STU00215236). The research was conducted according to the Declaration of Helsinki.

## 3 Participant group

**Figure 1:**
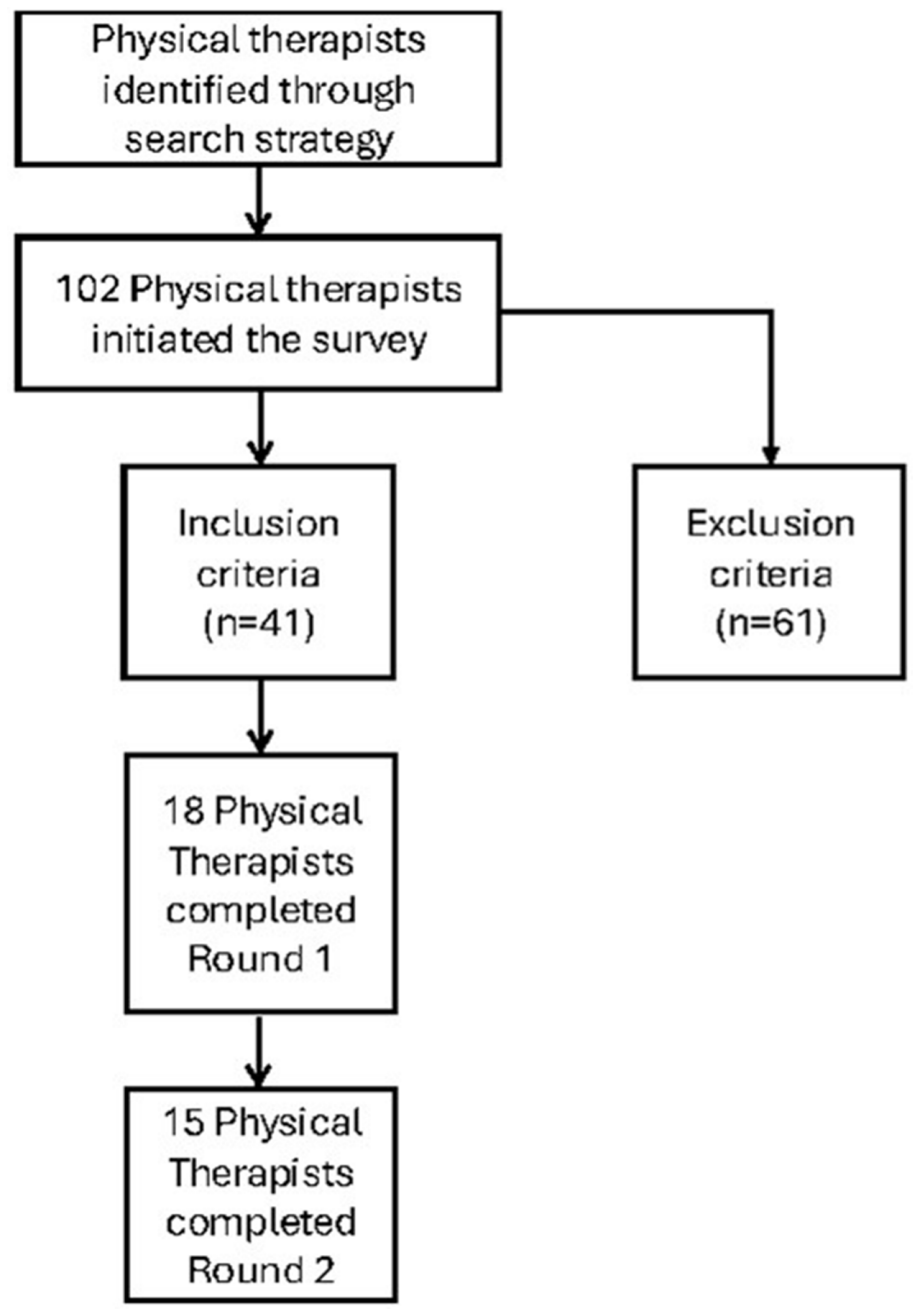
Participant selection process.

Participant recruitment for the survey was conducted via physical therapy Facebook groups, email list serves and the American Physical Therapy Association newsletter. Consideration was given to include other disciplines, but ultimately only physical therapists were included to assure the results were reflective of and applicable to physical therapy practice. To assemble a group of relative experts, strict inclusion criteria was established. In order to qualify for survey participation an individual had to be a physical therapist who applied casts specifically for spinal muscular atrophy. The participant qualification process is depicted in Figure 1. Many individuals (102) were able to initiate the survey but were unable to continue (*n* = 61) as they did not fit the criteria. The 18 individuals on the expert panel were included in the survey recruitment, but the 18 individuals who completed the survey were not solely the individuals on the expert panel. The expert panel included individuals who were knowledgeable in the field of SMA, but all were not involved in serial casting.

Prior to finalizing consensus, members of the SMA community who had participated in at least one bout of serial casting were recruited to add patient and family input to the recommendations. Community members were recruited to participate in semi structured interviews based on recommendations from the expert panel. Interest in participation was confirmed by the study’s investigators, with one secondary investigator completing the interview. A total of three interviews were conducted, with representatives from different programs throughout the United States: Pennsylvania; Ohio; and California. All programs were based in large, metropolitan areas.

## 4 Procedures

For all Delphi rounds, the participants received an invitation by email with a link to an online REDCap questionnaire. The participants were initially given 12 weeks to complete each round with reminders emailed weekly and more frequently near the end of survey collection. The invitation and study objectives were provided prior to the initial survey with informed consent obtained in conjunction with survey initiation. In order to meet inclusion criteria, an individual had to select that they were a clinician who works with patients with SMA and were familiar with serial casting.

The first part of the questionnaire included a list of demographic questions. The second part of the survey was structured into 3 primary domains: clinical appropriateness; program considerations and program feasibility/adherence. Clinical appropriateness was defined as appropriateness of the individual being considered for casting and whether individual characteristics would lead to a positive casting outcome. Program-based considerations were defined as the qualities a successful casting program should include and what procedures should be followed. Feasibility/Adherence was defined as considerations to determine if an individual could feasibly participate in the program and adhere to the determined guidelines.

The clinical appropriateness domain included sub-categories of consideration for function, range of motion, alignment, strength, timing, patient characteristics and outcome measures. The program-based considerations domain included sub-categories of timing, skin tolerance, functional considerations, patient characteristics, program follow-up, alignment factors when casting, home exercise recommendations, goals, patient/caregiver experience, and post-casting recommendation. The program adherence/feasibility domain included determining factors for adherence/buy-in, compliance factors, patient/caregiver education, staffing, reimbursement and scheduling.

These categories and the questions that fell under each of them were initially provided by the lead site and revised by the expert panel after deliberation and consideration in three pre-survey meetings. For the first round of the Delphi study, participants were asked to select “agree” or “disagree” for each of the questions. When a participant disagreed, a text box was provided to explain rationale. Additionally, for the first survey round, at the end of each category a participant was asked if any questions were redundant and, if so, which were thought to be repetitive. This information was then considered when updating the survey for the second round.

The level of agreement among the experts was analyzed dichotomously by means of average agreement. Agreement of > = 75% was considered reflective of group consensus as recommended for Delphi studies (23).

For the second-round survey, participants who completed the initial survey were recruited and informed of the results from the initial round. The second round of questions again included potential selections of agree or disagree with one more open-ended question regarding definition of plateau.

For the semi-structured interviews, recordings were synthesized with themes drawn and recorded. These themes and notes were then compared for consistency to the recommended guidelines. When consistency was not present, additional considerations and notes were added to the guidelines.

### 4.1 Data analysis

Analyses were performed using Excel and R Studio to obtain consensus. For each question, a total percentage of agreement was reached. Based on a paper by Keeney et al. (24), strong consensus was defined as anything above 90%, general consensus was defined as above 75% and lack of consensus was defined as less than 75% agreement.

Excel and R studio were also used to analyze participant demographics. RedCAP software was used to assess the standard deviation and frequency of the nominal variables.

## 5 Results

After two survey rounds conducted between June 2022 and March 2023, a consensus was reached among expert clinicians in all three areas proposed for the serial casting domains.

In the initial round, 103 individuals initiated the survey. Of those individuals, 61 qualified to complete the survey based on the specific inclusion criteria of experience in serial casting in SMA. Forty-five participants provided consent and agreed to participate with 18 participants fully completing all components of the first survey round for a response rate of 40%. Most (*n* = 14, 78%) of the 18 participants who fully completed all components indicated having over 15 years of experience as a practicing clinician and over half had completed doctoral degrees. Most participants also indicated having between 0 and 5 years of experience with serial casting (*n* = 7) and 0–5 years of experience casting neuromuscular disorders (*n* = 7). Two countries and 8 states from the US were represented in the study. Full demographic information for the participants is provided in Table 1.

**Table 1:** Demographics.

	Round 1	Round 2
Geography		
Canada	1	1
India	1	0
UK	1	1
USA	15	13
Highest level of education obtained		
Certificate	1	0
BS	4	3
MS	4	3
DPT	10	9
Additional Doctorate	0	0
Years as a practicing clinician		
0–5	1	0
6–10	1	1
11–15	2	1
>15	14	13
Primary discipline		
PT	17	15
OT	0	0
Orthotist	1	0
Other	0	0
Areas involved (past 3 years)		
Academic—Teaching	6	5
Academic—Research	5	4
Industry Clinical Trials	6	6
Clinical Trial Evaluation	8	8
Multidisciplinary Evaluation/Management Clinic for SMA	10	8
Outpatient treatment of SMA	12	10
Inpatient Treatment of SMA	8	5
Day Rehabilitation	2	1
Early Intervention	6	5
School System	1	0
% of Caseloads w/ Neuromuscular Disorders		
0–25%	8	7
26–50%	4	3
51–75%	1	1
76–100%	5	4
SMA specific questions		
Ages of Individuals w/ SMA Worked With		
Infants (<2 years)	14	11
Toddlers (2–4 years)	16	13
Children (>4–10 years)	15	14
Adolescents (>10–18 years)	12	11
Young Adults (>18–40 years)	8	7
Adults (>40 years)	2	2
Phenotypes worked with		
Presymptomatic	12	10
Non-sitters	14	12
Sitters	16	14
Walkers	14	13
Adults—onset in childhood	4	4
Adults—onset in adulthood	1	1
Casting specific questions		
Years of experience with serial casting		
0–5	7	5
6–11	1	1
12–20	5	4
>20	5	5
Years of experience casting neuromuscular disorder		
0–5	7	6
6–10	3	2
11–15	6	2
>15	5	5
How much of casting time is occupied by neuromuscular individuals		
0–25%	14	12
> = 26–50%	2	1
> = 51–75%	0	0
> = 76–100%	2	2
Of those neuromuscular serial casting encounters, how many individuals have SMA		
0–25%	14	11
> = 26–50%	2	2
> = 51–75%	2	2
> = 76–100%	0	0
Joints casted for SMA		
Elbow	1	0
Wrist	1	0
Knee	8	7
Ankle	17	15
Other	1	0
Serial casting for non-SMA diagnosis		
Yes	16	13
No	2	2
Diagnoses (for question above)		
Cerebral palsy	15	12
Muscular dystrophy	12	11
Arthrogryposis	5	2
Idiopathic toe walker	5	4
Other	4	1
Who does serial casting for SMA at facility		
Physician	2	1
Ortho tech	5	4
Orthotist	5	4
PT	14	14
OT	0	0

Fifteen participants fully completed all components of the second survey round for a response rate of 83.3%. Most (*n* = 13, 86.6%) of the 15 participants who fully completed all components indicated having over 15 years of experience as a practicing clinician. Most participants also indicated having between 0 and 5 years of experience with serial casting (*n* = 5) or over 15 years of experience casting (*n* = 5) and 0–5 years of experience casting neuromuscular disorders (*n* = 5).

For round two, a revised list of questions in the areas of non-agreement were distributed to the initial 18 respondents. In this round, 15 surveys (83.3%) were completed. Responses were reviewed with 96.6% agreement for the revised questions on clinical appropriateness, 95.0% for program-based considerations and 96.86% for program adherence/feasibility.

At the end of two rounds, a total of 296 items reached consensus. These items were distributed among three primary domains: clinical appropriateness (*n* = 184), program considerations (*n* = 124) and feasibility/adherence (*n* = 88).

For *Clinical Appropriateness*, in round one, 3% of the items did not reach consensus. There were six new questions proposed and in Round two, all items reached consensus.

During round one, participants’ concerns focused around being able to maintain transfer ability and function. Specifically, concerns about the weight of the cast and alternative methods of transport were mentioned as reasons for disagreement. For loss of function, participants raised concerns about there being a decrease in function when a cast is first donned that is later recovered. In round two, all four of the question modifications in the Clinical Appropriateness Domain made after Round 1 obtained consensus, although in some situations a participant would disagree with a statement on the basis of not having encountered the situation in their experience and practice (*n* = 1) or misinterpretation of the question (*n* = 2) regarding expected functional level and the need for patient specificity.

For *Program Based Considerations*, in round one, 9% of the items (*n* = 16) did not reach consensus. There were four question modifications proposed and in round two, all items (*n* = 4) reached consensus.

In round one, participants disagreed with the time of casting, the type of cast used, and casting goals. For the time, participants mentioned longer or shorter casting time from the proposed 7–8 weeks. In round two, the proposed question modification specified casting until reaching a plateau which achieved consensus. The type of cast questions was excluded, since multiple participants (*n* = 5) did not have experience with the two types of boots [CAM boot (3) and AFO braces (2)] and the question did not reach consensus. Lastly, casting degree goals were switched to being patient specific, which all but one participant agreed with, on the basis that all decisions in casting should be patient specific.

For *Program Adherence/Feasibility*, in round one, 24% of the items did not reach consensus. There were two new questions proposed in round two, and all items then reached consensus. Most of the questions that failed to reach consensus involved who could perform serial casting and who could do the billing. Most participants felt that a physical therapist should be performing the casting and subsequently doing the billing. The two new questions constructed from this feedback stated that a physical therapist should be present during the casting and that billing should be done by the person who performed the casting. Both questions reached consensus, and only one participant disagreed with the casting question on the basis that the orthotists could do the casting if they had sufficient experience.

Common themes emerged in the semi-structured interviews. These included the need for clear expectations and education prior to casting (likely functional performance in casts including potential impact to sleep, bathing considerations, common tools and strategies for maintaining comfort, process for cast removal, potential for pain, definition of frequently used terms, addressing both typical and atypical responses, need for skin protection and practice of safe falling), considerations for timing and feasibility in regard to patient age, family schedule and emergency preparedness. In congruence with the guidelines, all individuals interviewed had participated in casting with a clear functional goal and underwent casting for a predetermined period of time and all highly recommended casting be conducted by a trained individual with inclusion of a physical therapist on the team. Some discrepancies from the draft recommendations were noted regarding who was performing the casting and their training background, exercise recommendations during and post-casting, and the extent of post-casting follow-up.

Some interviewees noted a significant benefit to casting. Another spoke of its potential to prolong an inevitable need for surgery. Another described the process as being a time-consuming and poor experience overall, but noted it being potentially more beneficial for an older child. One individual performed casting multiple times and noted a variability in techniques between providers but felt that having an expected casting routine provided good support to contracture management and maintained range in the interest of optimizing functional performance in a stander. Overall, the advantages of casting could be realized when standards were defined, casting methods were sound, and comfort and performance were given first priority.

**Figure 2:**
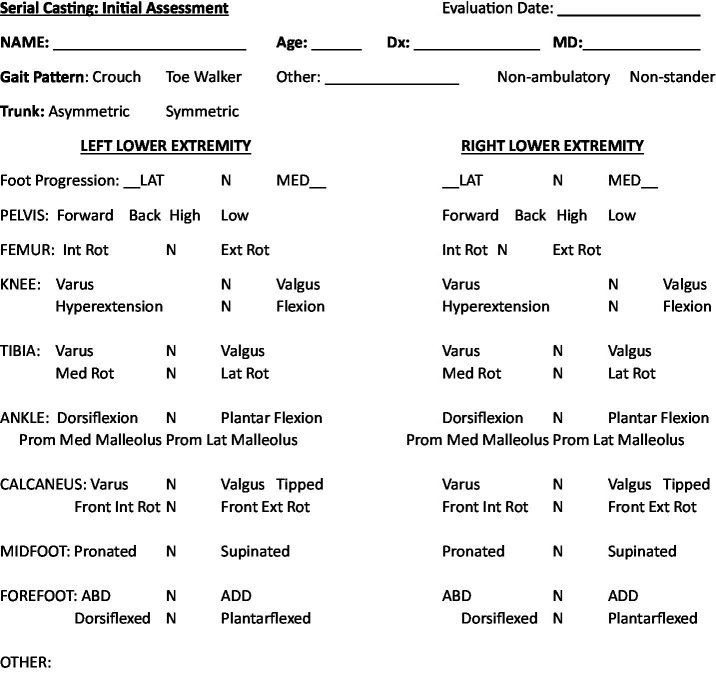
Recommended alignment assessment for initial serial casting.

The final list of critical information for serial casting in SMA, derived from consensus, for each domain was modified once community input was added and can be found in Appendix and Figure 2.

## 6 Discussion

The presence of contractures in SMA is a known and on-going concern even with the advent of disease-modifying therapies. When contractures are present, they can significantly limit an individual’s functional performance. Serial casting is one strategy to address contractures though there are no specific guidelines or evidence to guide this process with this specific diagnosis. Prior to collecting evidence to better assess the use of serial casting in SMA, a consensus approach to achieve guidelines for casting was undertaken using a modified-Delphi with additional validated information gained from semi-structured interviews. The Delphi approach is considered the most valid for obtaining expert opinions and given its widespread use in health science research, the information obtained is considered valuable in establishing the recommended guidelines. Additionally, the consideration of the community perspective gained through semi-structured interviews ensures that the established guidelines are meaningful and feasible to the impacted population. This insight of real and lived experience serves to validate and crosscheck the viewpoint of the clinician.

To establish guidelines in a survey format, it is necessary that participants have an appropriate level of experience and background treating individuals with SMA and in use of serial casting while also being representative of a variety of programs and locations. After analyzing the respondents, their years of clinical experience treating neuromuscular disease, years of experience in casting and diversity of background regarding geographic location, the results from the survey are considered reflective of expert opinion with a generalizability across regions. While other countries are represented in the survey, a majority of respondents were from the United States. Due to the high variability noted throughout healthcare systems internationally, areas such as billing and reimbursement were intentionally less specific.

The three domains surveyed reached a minimum of 75% consensus and thereby created a comprehensive guide to the different elements of serial casting considered critical for this population.

The comparison of the guidelines with semi-structured interviews provided valuable, real-world information and generally validated the previously established recommendations. However, the interviews noted some specific areas to highlight with the guidelines modified to increase the emphasis on and elaborate the components to be included in pre-casting education. Further, additional comments were added regarding the feasibility consideration for age not just from the perspective of range gained, but from the overall maturity standpoint and additional comments were added for timing to include consideration for family burden and impact. The interviews reinforced the need for increased consistency in training and background of individuals performing casting and for post-casting recommendations. This recommendation is consistent with the published guidelines for serial casting in the lower extremity recommending that therapists be appropriately educated, trained in competent in casting procedures (19). The difference in overall impact and casting experience provides helpful background related to patient and caregivers and acknowledges the lived experience in a manner that the clinician generated guidelines could not. With this information supplementing the guidelines, the resulting document is felt to be comprehensive, feasible and reflective of a detail necessary to optimize the casting experience.

The established guidelines should be shared using education and knowledge translation to establish feasibility and implementation/uptake of use. Utilization and application by individuals involved in serial casting programs involving individuals with SMA is recommended. Future work should aim at using these guidelines as a platform for assessing the efficacy of serial casting to improve and maintain range as well as influence function in those with SMA. Collecting both single and multi-site data using the recommended guidelines in a standardized format could ultimately inform efficacy of use across the spectrum of those with SMA. Translation of the guidelines into different languages with global access and distribution should occur over time. It should be noted that while other countries are represented on the expert panel, there are limited survey respondents from countries outside of the US. This limits the overall generalizability and should be considered as guidelines are applied.

There are certain limitations to acknowledge that may have affected the ultimate outcome of the survey. The number of responses were limited and may have been a result of the initial length of the survey, limited number of professionals involved with both serial casting and treating those with SMA or influences of the response platform utilized. Additionally, these guidelines are limited to physical therapy experience and input. Other practitioners who participate in serial casting offer valuable insights, but it is the consensus of the group that as experts in movement, a physical therapist should always be involved. Future surveys could further consider insights of other provider types. Limitations to consider in the semi-structured interviews were the small number of participants and are that all programs represented were from large and metropolitan areas. This may have biased the standardization of procedures and background of the individuals performing the casting.

Finally, future work should aim at establishing guidelines for the upper extremity, as well. Casting for the elbow joint has been frequently reported in other disorders and can result in considerable functional gains. Therefore, the guidelines could be expanded to include upper extremity application as well.

After expert panel discussions, a two-round Delphi survey and semi-structured interviews with community participants regarding serial casting in SMA, there were specific areas of consensus in the domains of clinical appropriateness, program-based considerations and program adherence/feasibility. Guidelines for serial casting in SMA have been established to provide a platform for future research and consideration in contracture management. This framework can be used to guide, create, and revise casting programs for those with SMA.

## Data Availability

The raw data supporting the conclusions of this article will be made available by the authors, without undue reservation.

## References

[1] Berciano MT Castillo-Iglesias MS Val-Bernal JF Lafarga V Rodriguez-Rey JC Lafarga M. Mislocalization of SMN from the I-band and M-band in human skeletal myofibers in spinal muscular atrophy associates with primary structural alterations of the sarcomere. Cell Tissue Res. (2020) 381:461–78. doi: 10.1007/s00441-020-03236-3, PMID: 32676861

[2] Salazar R Montes J Dunaway Young S McDermott MP Martens W Pasternak A. Quantitative evaluation of lower extremity joint contractures in spinal muscular atrophy: implications for motor function. Pediatr Phys Ther. (2018) 30:209–15. doi: 10.1097/PEP.0000000000000515, PMID: 29924070

[3] Skalsky AJ McDonald CM. Prevention and management of limb contractures in neuromuscular diseases. Phys Med Rehabil Clin N Am. (2012) 23:675–87. doi: 10.1016/j.pmr.2012.06.009, PMID: 22938881 PMC3482407

[4] Walker MP Rajendra TK Saieva L Fuentes JL Pellizzoni L Matera AG. SMN complex localizes to the sarcomeric Z-disc and is a proteolytic target of calpain. Hum Mol Genet. (2008) 17:3399–410. doi: 10.1093/hmg/ddn234, PMID: 18689355 PMC2566527

[5] Jha NN Jk K Her YR Monani UR. Muscle: an indepedent contributor to the neuromuscular spinal muscular atrophy disease phenotype. JCI Insight. (2023) 8:1–11. doi: 10.1172/jci.insight.171878, PMID: 37737261 PMC10561723

[6] Rajendra TK Gonsalvez GB Walker MP Shpargel KB Salz HK Matera AG. A drosophila melanogaster model of spinal muscular atrophy reveals a function for SMN in striated muscle. J Cell Biol. (2007) 176:831–41. doi: 10.1083/jcb.200610053, PMID: 17353360 PMC2064057

[7] Kim JK Jha NN Feng Z Faleiro MR Chiriboga CA Wei-Lapierre L. Muscle-specific SMN reduction reveals motor neuron-independent disease in spinal muscular atrophy models. JCI. (2020) 130:1271–87. doi: 10.1172/JCI131989, PMID: 32039917 PMC7269591

[8] Williams PE Goldspink G. The effect of denervation and dystrophy on the adaptation of sarcomere number to the functional length of the muscle in young and adult mice. J Anat. (1976) 122:455–65. 1002614 PMC1231915

[9] Glanzmann A Gardner M Musey N Flickinger J Jones J Evans SH. Serial casting for contracture in SMA a comparative analysis with cerebral palsy and duchenne muscular dystrophy. Orlando, FL: CureSMA (2023).

[10] Glanzman AM Flickinger JM Dholakia KH Bönnemann CG Finkel RS. Serial casting for the management of ankle contracture in Duchenne muscular dystrophy. Pediatr Phys Ther Fall. (2011) 23:275–9. doi: 10.1097/PEP.0b013e318227c4e3, PMID: 21829124 PMC5210185

[11] Hosl M Bohm H Arampatzis A Doderlein L. Effects of ankle-foot braces on medial gastrocnemius morphometrics and gait in children with cerebral palsy. J Child Orthop. (2015) 9:209–19. doi: 10.1007/s11832-015-0664-x, PMID: 26108740 PMC4486505

[12] Weppler CH Magnusson P. Increasing muscle extensibility: a matter of increasing length or modifying sensation? Phys Ther. (2010) 90:438–49. doi: 10.2522/ptj.20090012, PMID: 20075147

[13] Hof AL. Changes in muscles and tendons due to neural motor disorders: implications for therapeutic intervention. Neural Plast. (2001) 8:71–81. doi: 10.1155/NP.2001.71, PMID: 11530889 PMC2565391

[14] Tardieu C Tabary JC Tabary C Huet De La Tour E. Comparison of the sarcomere number adaptation in young and adult animals. Influence Tendon Adaptation J Physiol. (1977) 73:1045–55. PMID: 615249

[15] Tabary JC Tabary C Tardieu C Tardieu G Goldspink G. Physiological and structural changes in the cat's soleus muscle due to immobilization at different lengths by plaster casts. J Physiol. (1972) 224:231–44. doi: 10.1113/jphysiol.1972.sp009891, PMID: 5039983 PMC1331536

[16] Carroll K de Valle K Kornberg A Ryan M Kennedy R. Evaluation of serial casting for boys with Duchenne muscular dystrophy: a case report. Phys Occup Ther Pediatr. (2018) 38:88–96. doi: 10.1080/01942638.2017.1280874, PMID: 28300461

[17] Main M Mercuri E Haliloglu G Baker R Kinali M Muntoni F. Serial casting of the ankles in Duchenne muscular dystrophy: can it be an alternative to surgery? Neuromuscul Disord. (2007) 17:227–30. doi: 10.1016/j.nmd.2006.12.002, PMID: 17303425

[18] Rose KJ Raymond J Refshuage K North K Burns J. Serial night castng increases ankle dorsiflexion range in children and young adults with Charcot-Marie-tooth disease: a randomised control trial. J Physiother. (2010) 56:113–9. doi: 10.1016/S1836-9553(10)70041-2, PMID: 20482478

[19] Cincinnati Children’s Hospital Medical Center Health Policy and Clinical Effectiveness Program. (2009). Evidence-based care guideline for management of serial casting in children. Available at: https://www.google.com/url?sa=t&rct=j&q=&esrc=s&source=web&cd=&ved=2ahUKEwjr_sSt6uyCAxW6GDQIHdJQCTIQFnoECA0QAQ&url=https%3A%2F%2Fwww.cincinnatichildrens.org%2F-%2Fmedia%2FCincinnati-Childrens%2FHome%2Fservice%2Fo%2Fot-pt%2Fserial-casting%2FSerial-Casting-Guideline.pdf&usg=AOvVaw2wrGoFYMdORi2ESmEiZbO6&opi=89978449 (Accessed November 30, 2023)

[20] Milne N Miao M Beattie E. The effects of serial casting on lower limb function for children with cerebral palsy: a systematic review with meta-analysis. BMC Pediatr. (2020) 20:324. doi: 10.1186/s12887-020-02122-9, PMID: 32615954 PMC7330971

[21] d’Astrog H Rampal V Seringe R Glorion C Wicart P. Is non-operative management of childhood neurologic cavovarus foot effective? Orthop Traumatol Surg Res. (2016) 102:1087–91. doi: 10.1016/j.otsr.2016.09.006, PMID: 27825708

[22] Tachdjian MO. *The child’s foot* (1^st^ ed.). Philadelphia: WB Saunders Company (1985).

[23] Niederberg M Koberich S, Members of the DeWiss Network. Coming to consensus: the Delphi technique. Eur J Cardiovasc Nurs. (2021) 20:692–5. doi: 10.1093/eurjcn/zvab059, PMID: 34245253

[24] Keeney S Hasson F HP MK. A critical review of the Delphi technique as a research methodology for nursing. Int J Nurs Stud. (2001) 38:195–200. doi: 10.1016/S0020-7489(00)00044-4 11223060

[25] Fujak A Kopschina C Gras F Forst R Forst J. Contractures of the lower extremities in spinal muscular atrophy type II. Descriptive clinical study with retrospective data collection. Ortop Traumatol Rehabil. (2011) 13:27–36. doi: 10.5604/15093492.933792, PMID: 21393646

