# Correction: Serial casting for contractures in SMA: consensus derived guidelines for treatment

**DOI:** 10.3389/fneur.2025.1646078

**Published:** 2025-07-16

**Authors:** Laurey Brown, Katie Hoffman, Chiara Corbo-Galli, Carolyn Kelley, Terri Carry, Matt Civitello, Giorgia Coratti, Roberto DeSanctis, Tina Duong, Brigid Driscoll, Jean Flickinger, Allan M. Glanzman, Jennifer Jones, Elizabeth Maczek, Dionne Moat, Jacqueline Montes, Robert Muni-Lofra, Leslie Nelson, Amy Pasternak, Melanie Valle, Kristin J. Krosschell

**Affiliations:** ^1^Ann & Robert H. Lurie Children's Hospital of Chicago, Chicago, IL, United States; ^2^Weinberg College of Arts and Sciences, Northwestern University, Evanston, IL, United States; ^3^Children's Hospital of Colorado, Aurora, CO, United States; ^4^St. Jude Children's Research Hospital, Memphis, TN, United States; ^5^Agostino Gemelli University Hospital, Rome, Italy; ^6^Department of Neurology, Stanford University, Stanford, CA, United States; ^7^Children's Hospital of Philadelphia, Philadelphia, PA, United States; ^8^Boston Children's Hospital, Boston, MA, United States; ^9^Newcastle upon Tyne Hospitals NHS Foundation Trust, Newcastle upon Tyne, United Kingdom; ^10^Columbia University Irving Medical Center, New York, NY, United States; ^11^Dallas Children's Medical Center, Dallas, TX, United States; ^12^Department of Physical Therapy, University of Texas – Southwestern, Dallas, TX, United States; ^13^Northwestern University Feinberg School of Medicine, Chicago, IL, United States

**Keywords:** Delphi technique, consensus, spinal muscular atrophy, contracture, serial casting

In the published article, some affiliations were listed incorrectly. Robert Muni-Lofra was mistakenly affiliated with Agostino Gemelli University Hospital, Rome, Italy, and Roberto DeSanctis with Newcastle Upon Tyne Hospitals NHS Foundation Trust, Newcastle upon Tyne, United Kingdom.

The correct affiliation is as follows:

Robert Muni-Lofra should be affiliated with Newcastle Upon Tyne Hospitals NHS Foundation Trust, Newcastle upon Tyne, United Kingdom, and Roberto DeSanctis with Agostino Gemelli University Hospital, Rome, Italy.

The original version of this article has been updated.

