# Supplementary material for: Serial casting for contractures in SMA: consensus derived guidelines for treatment
Source: Front Neurol. 2025 Apr 16;16:1502495. doi: 10.3389/fneur.2025.1502495 (PMC12040694; doi:10.3389/fneur.2025.1502495)
Supplement: Supplementary file 1 [file Data_Sheet_1.pdf]

<u>Serial Casting: Initial Assessment</u> Evaluation Date: ________________

NAME: ________________________ **Age:** ______ **Dx:** ________________ **MD:**_______________

Gait Pattern: Crouch Toe Walker Other: _________________ Non-ambulatory Non-stander

Trunk: Asymmetric Symmetric

	**<u>LEFT LOWER EXTREMITY</u>**			**<u>RIGHT LOWER EXTREMITY</u>**		
Foot Progression:	__LAT	N	MED__	__LAT	N	MED__
PELVIS:	Forward Back High	Low		Forward Back High	Low	
FEMUR:	Int Rot	N	Ext Rot	Int Rot	N	Ext Rot
KNEE:	Varus	N	Valgus	Varus	N	Valgus
	Hyperextension	N	Flexion	Hyperextension	N	Flexion
TIBIA:	Varus	N	Valgus	Varus	N	Valgus
	Med Rot	N	Lat Rot	Med Rot	N	Lat Rot
ANKLE:	Dorsiflexion	N	Plantar Flexion	Dorsiflexion	N	Plantar Flexion
	Prom Med Malleolus	Prom Lat Malleolus		Prom Med Malleolus	Prom Lat Malleolus	
CALCANEUS:	Varus	N	Valgus Tipped	Varus	N	Valgus Tipped
	Front Int Rot	N	Front Ext Rot	Front Int Rot	N	Front Ext Rot
MIDFOOT:	Pronated	N	Supinated	Pronated	N	Supinated
FOREFOOT:	ABD	N	ADD	ABD	N	ADD
	Dorsiflexed	N	Plantarflexed	Dorsiflexed	N	Plantarflexed

OTHER:
