# Supplementary material for: Serial casting for contractures in SMA: consensus derived guidelines for treatment
Source: Front Neurol. 2025 Apr 16;16:1502495. doi: 10.3389/fneur.2025.1502495 (PMC12040694; doi:10.3389/fneur.2025.1502495)
Supplement: Supplementary file 2 [file Data_Sheet_2.pdf]

Table 2.

Guidelines to Serial Casting in Spinal Muscular Atrophy

1. Clinical Appropriateness.

Prior to beginning a casting program, it is essential to consider if an individual is appropriate to be cast. The following considerations are considered paramount.

- 1.1 Emphasis on function:

  A potential for functional gain or for the maintenance of a current functional ability is the primary rationale for performing serial casting. A change in range of motion without a meaningful outcome (i.e., gait quality, wheelchair positioning, pain, etc…) would not be a worthwhile endeavor.

  Gains in function will vary based on an individual's functional level at the initiation of serial casting. For ambulatory individuals, casting should be considered if looking to improve or maintain efficiency and quality of gait as well as to prolong ambulation status and capability. For non-ambulatory individuals, casting should be considered in regard to participation in a standing program focusing on the opportunity to gain or maintain current ability either with a stander or for performing unassisted standing using Knee Ankle Foot Orthotics (KAFOs).

  - 1.1.1 Prior to casting, a person's ability to transfer with casts donned should be considered. While it is possible a person's transfer ability will be impaired while using casts, a return to prior transfer ability following casting should be expected. Additionally, for ambulatory individuals, consideration should be given to the fact that compensatory movements without casts may impact a person's previous level of function and reveal areas of impairment that were previously considered masked. (See list for comprehensive list of transfers/skills to consider.)
  - 1.1.2 If, due to typical disease course and development of contractures, a patient is nearing the loss of ambulation or other functional mobility and more immediate and timely assessment for casting should be considered.
  - 1.1.3 Provided the evolving landscape with drug therapy, clinicians should consider that individuals may have more potential to improve or re-gain function than previously demonstrated with prior natural history. For a person who has initiated drug therapy and previously deemed not appropriate for casting, it's possible that casting could be reconsidered and assessed for potential benefit and functional gain.
  - 1.1.4 It is reasonable to consider repeat casting on the same joint when an individual demonstrates a change in function and casting may provide an opportunity to return to a previous functional level.

<table>
<tr><th>Considerations for Non-Ambulators</th><th>Considerations for Ambulators</th><th>Considerations for all functional levels during casting</th></tr>
<tr><td rowspan="2">Positioning in assistive device</td><td>Ease of ambulation due to weight of cast</td><td>Bathing</td></tr>
<tr><td>Concern for falls and injury</td><td>Wheelchair with elevating leg rests</td></tr>
<tr><td rowspan="3">Self-care</td><td rowspan="3">Potential to acquire, improve or maintain current level of ambulation</td><td>Transfers</td></tr>
<tr><td>Bed Mobility</td></tr>
<tr><td>Sleeping</td></tr>
<tr><td colspan="2" rowspan="2"></td><td>Stairs (home and school)</td></tr>
<tr><td>Pain</td></tr>
</table>

- 1.2 Range of Motion Factors:

  Prior to performing casting, all of the following range of motion measurements should be acquired with recommendation for specific positioning for consistency and accuracy.

**Joint Measurement**	**Position**
Ankle Dorsiflexion with Knee Flexed	Prone*. Using subtalar joint (STJ) neutral with knee flexed, head down and hands at sides
Ankle Dorsiflexion with Knee Extended	Prone*. Using STJ neutral with knee extended, head down and hands at sides.
Calcaneal inversion/eversion	Prone. Head down and hands at side.
Knee Extension	Supine with neutral LE alignment
Popliteal Angle	Supine with opposite lower extremity in neutral hip and knee extension
Hip extension	Side-lying with trunk in neutral position

*If a patient is unable to achieve a prone position, supine with maximal available knee extension is considered the next best position.

- 1.3 Parameters:

  There are 3 primary scenarios that would yield consideration for initiation of a serial casting program. Clinical judgment should be applied when pursuing these scenarios. Please refer to the algorithm for additional information.
    - 1) An individual has demonstrated a failed attempt in the use of night splints to maintain adequate range at the ankle. This failure can include both the inability to wear the devices or a lack of compliance.
    - 2) An individual has demonstrated failure of manual stretching and/or home program execution to maintain adequate or improved dorsiflexion range at the ankle. This failure can include a lack of compliance.
    - 3) For a non-ambulatory person, when the ability to attain a neutral foot position for transfers, positioning and mobility devices is not possible and AFOs, stretching and home programs have been deemed ineffective.

- 1.4 Alignment Factors:

  Alignment is an important consideration both before and during casting. A comprehensive list of alignment considerations is listed below. Prior to initiating a casting program, a clinician should weigh the risks and benefits of an individual's function in poor alignment versus a decline in function for good alignment.
    - Alignment factor check-list: See Figure 2 for evaluation form for use with patients.
        - Midfoot and forefoot mobility and alignment
        - First ray alignment
        - Forefoot to rearfoot alignment, specifically (forefoot varus/valgus and forefoot abduction/adduction
        - Postural asymmetries
        - Presence and impact of scoliosis on alignment
        - Position of thorax relative to pelvis (anterior/posterior/neutral) (ambulatory only)
        - Pelvic orientation in all planes (rotation, obliquity, posterior or anterior pelvic tilt)
        - Femoral position (neutral, internal/external rotation)
        - Knee valgus/varus
        - Knee hyperextension/flexion
        - Tibia alignment (presence of tibial torsion)
        - Subtalar Neutral Alignment
        - Medial and Lateral malleolus prominence
        - Calcaneal varus/valgus or tipped orientation
        - Foot progression angle
        - Presence of joint hypermobility

- 1.5 Strength Considerations:

  Underlying strength and ability for generating strength about a joint should be considered prior to and during casting. The following muscle tests are recommended prior to initiation of a casting program:
  - Hip Flexion (Iliopsoas)
  - Anterior Tibialis
  - Posterior Tibialis
  - Peroneus Brevis/Longus
  - Extensor Digitorum Longus
  - Gastrocnemius (via standing or hand test)
    - Calf circumference should be obtained prior to initiation and throughout casting process in order to help monitor for atrophy

- 1.6 Timing Considerations:

  Some additional components regarding best timing should be considered prior to initiation of casting.

  - The outdoor climate of the geographic area as well as any climates for potential vacations. Casting can be difficult to tolerate in very warm environments. When seasons offer opportunities for more comfortable weather and casting at this time is a possibility, it should be taken into consideration.
  - If a patient is to begin or is scheduled for a routine dosage of disease modifying therapies. The dosing schedule should be taken into consideration along with timing the initiation of a casting program.
  - A person's age must also be taken into consideration with younger ages typically demonstrating greater gains in casting. There is no known maximum age for the ability to make changes in range.
  - It is reasonable to repeat casting on the same joint if ROM has been lost during a growth spurt.
  - If a person has previously demonstrated successful improvements, it is reasonable to repeat casting on the same joint in order to regain range.
  - Early referral or attempt to cast earlier in the process of contracture development are associated with increased pliability in contractures and a greater likelihood of improved results.

- 1.7 Patient Characteristics:

  Different patient characteristics also play into the potential efficacy of a casting program. The following should be considered prior to initiation:

- Sensory concerns (i.e., inability to tolerate cast saw sound, different textures of casting materials and tolerance to pressure applied to foot/ankle that may impede the casting process
- Vascular or Lymphatic health and potential for increased swelling/sweat response
- Though seen more frequently with the use of Thoracolumbar Sacral Orthotics (TLSOs), consideration should be given to potential for autonomic storming as a result of wearing casts
- Gait observations and notation of gait asymmetries, when applicable
- Ability to bear weight through bilateral LEs

- 1.8 Outcome measures:

  There are a variety of outcome measures to consider when tracking individual outcomes for serial casting. Some are more relevant for the ambulatory population though a conglomerate of measures should be considered for use with either functional status. The following are considered relevant to a casting program and should be performed (when applicable due to function) 2-4 weeks prior to casting and then re-evaluated 2-4 weeks post-casting.

Outcome Measure	Rationale
6 MWT	To assess ambulation and any biomechanical changes with fatigue
Peds QL	Assess quality of life
PediCAT	Assess quality of life
Timed Tests of Function: Supine to stand; 10m walk/run; 4 stair climb	To quantify functional performance and track changes
ROM Measurements	To track changes
Balance (Static and Dynamic)	To monitor safety and stability

2. *Program Based Considerations*

Once a person is considered appropriate for casting, there are a number of considerations and recommendations regarding the ideal program. Below is a guide regarding timing, alignment factors, skin tolerance, functional considerations, goals, home exercise recommendations, patient/caregiver experience, post-casting recommendations and program follow-up.

- 2.1 Donning Casts:

    - Consistent with literature in the neuromuscular population (Weppler), a person should be cast at R1 in order to not over-stretch and preserve changes in the length/tension relationship as well as ground reaction forces.

- 2.2 Timing: The following timing recommendations are considered standard for casting programs.
    - Typical casting programs last anywhere from 3-8 weeks.
    - Serial casts are changed at least 1x/week.
    - If needed, a casting course can be broken into 2 segments with 6 weeks off in between.
    - If intending to cast knees and ankles, the ankles should be cast first and then the contribution of the knees determined.
        - When casting for knee flexion contractures, some clinicians have observed a new development of quadriceps contractures. It is recommended that quadricesp stretching be done between each cast to ameloriate potential effect [9]
    - Casting is terminated based on therapist interpretation of performance on weekly assessment and indicated when a person demonstrates a lack of ROM progression or plateau, pain, and inability to maintain functional performance.

- 2.3 Recommended casting materials:There are many acceptable materials to utilize during casting. When appropriate and when indicated, waterproof cast protectors can be utilized as can waterproof casting materials. However, waterproof casts are, in some cases, more expensive than regular fiberglass casts and may not be covered by insurance. Additionally, some individuals may have increased skin sensitivity with the materials in the first layer of the waterproof cast. These considerations should be taken into account prior to initiating use of a waterproof cast. Delta casts are also noted to be lighter weight and may assist in maintaining functional performance when decreased strength is an overriding concern. There is no known difference in efficacy with the use of certain materials.

- 2.4 Alignment factors while in casts: It is recommended that a person always be cast bilaterally. Care should also be taken to level the pelvis. If ambulatory this is to be done in standing and if non-ambulatory, this can be achieved with the foot plates in the stander. When casts are being applied, the individual casting should align to subtalar joint neutral and the 1st ray and forefoot. And, neutral knee alignment should be emphasized and may be obtained by posting and lifts applied outside the casts or placed in casting boots.

- 2.5 Skin Tolerance: Skin should be monitored for breakdown throughout the casting progress. If a patient is demonstrating poor tolerance, the casting could be broken up into segments. In order to prevent pressure areas, discomfort or breakdown at bony prominences such as the first ray or lateral malleolus, extra padding in addition to cut windows using duoderm or gel pads to protect bony prominences should be used.

- 2.6 Functional Considerations: As stated in the section regarding the clinical appropriateness of the individual being cast, the functional gain should be foremost in mind. The person performing casting should be aware not to push the individual being casted past their functional range of motion and also be cognizant that improvement in alignment and effective muscle control has the potential to provide incremental functional gains.

- 2.7 Home Exercise Recommendations: The home program is considered a vital component for casting program success. The following activities are recommended for emphasis during casting: supported standing based on a person's previous amount of standing; walking program (when applicable) based on a person's previous amount of walking; general balance exercises that include those based on COM and postural control; seated weight-bearing and weight-shifting exercises for both ambulatory and non-ambulatory individuals.

- 2.8 Parent/Caregiver Education: Thorough education on the assessment of blood flow should be conducted as compromised circulation could become a concern.

- 2.9 Goals: As in most cases, goals should be individualized and emphasize function and participation. Casting program success can be illustrated when an individual demonstrates improvement on an outcome measure, pain reduction, achieves improved standing alignment or optimal positioning in a stander or KAFO (non-ambulatory) or by demonstrating improved gait mechanics (ambulatory).

- 2.10 Patient/Caregiver Experience: Assessing the experience of the patient and caregiver is a necessary element to the casting program. Areas of consideration include performing a survey including a Likert scale for patient tolerance; assessing quality of life; level of stress and burden to patient/caregiver; feasibility of program; and the degree to which expectations were met. These assessments should occur prior to the initiation of casting, during and at program completion.

- 2.11 Program Follow-up: Following completion of casting, it is recommended an individual have frequent visits (weekly or bi-weekly) either to outpatient PT or to serial casting clinic to monitor ROM and to modify or advance the Home Exercise Plan HEP. It is possible that casting may provide an opportunity to address underlying weakness and optimize mobility. If possible and indicated, consideration should be given to increase frequency of outpatient PT or more intensive rehab either during or following casting.
    - Ankel Foot Orthotic (AFO) use following casting is an integral element to maintaining the gained range. There are different recommendations based on an individual's functional status.
        - For non-ambulatory individuals,
            - Positional AFOs should be issued if utilizing a stander.
            - Night-time AFOs

        - Ambulatory Individuals:
            - Work with PT to determine if day-time AFO bracing is beneficial
            - Night-time AFOs
        - There are different types of AFOs to consider. Both ultraflex and solid AFOs are recommended. Use of an articulating AFO is not considered best practice.

3. *Program Adherence/Feasibility*

   It is important that a clinician take into account the likelihood of an individual to succeed with the casting program based on concepts related to adherence and feasibility. The following are provided as guides to determine the extent to which success might be considered.

   - 3.1 Factors for adherence and buy-in:
     - 3.1.1. When a patient has previously “failed” casting due to non-compliance, lack of follow through and family circumstances, it is not recommended that a patient be reconsidered to perform repeat casting on the same joint unless it is reasonable to conclude that a new episode of care would demonstrate a significantly different behavior or response.
     - 3.1.2 Prior to starting: The child and parent/caregivers desire to initiate a serial casting program should be taken into consideration.

   - 3.2 Compliance Factors: A clinician should take into account a patient’s maturity, behavioral presentation, social concerns in the home environment, lack of accountability to maintain care/safety/appointment follow-up.

   - 3.3 Patient/Caregiver Education: Thorough education is an important component to the success of the casting program. Education should include expectations prior to, during and following completion of a serial casting program (e.g., orthosis compliance post-casting, home program, during/post casting); anatomy and physiology; science behind casting; expected outcome; level of commitment (how often a patient will return for casting changes and how long return visits will be); and potential complications and issues that can arise during the casting episode.
     - Should concerns arise, parents and caregivers should be educated on where to go and who to call.
       - For weekdays, it is typical for casts to be removed by the department in which the casting was performed
       - For off hours and weekends, caregivers should be advised with indications for cast removal in the Emergency Department (ED) and should be provided with an emergency room letter.

- 3.4 Staffing: Staffing tends to be site and geography specific with serial casting conducted in a variety of scenarios including by PTs in the PT department and in conjunction with PT visits; in the orthotics department; by a casting technician; in the orthopedics department with an orthopedic technician. Similarly, support staff could include additional PTs; medical assistants, PT assistants, PT rehab aides and orthotists. However, due to expertise in movement and educational background, physical therapists are recommended to be involved at all elements of casting.

- 3.5 Reimbursement: Reimbursement differs based on geographic location, individual insurance providers and among programs. In general, the individual doing the casting should be listed as the billing provider.

- 3.6 Scheduling: There are a variety of acceptable practices for performing serial casting. Among these practices are within the rehab department and in a casting clinic. It is recommended that follow-up visits in either setting be allotted 1.5 to 2 hours to assure appropriate time is given to ensuring alignment, comfort in casts and patient/caregiver education.
